# Radiosynthesis and reactivity of *N*-[^11^C]methyl carbamoylimidazole

**DOI:** 10.1007/s10967-018-5948-4

**Published:** 2018-06-19

**Authors:** Manikandan Kadirvel, Déborah Cardoso, Sally Freeman, Gavin Brown

**Affiliations:** 1 Wolfson Molecular Imaging Centre, University of Manchester, Manchester, M20 3LJ UK; 2 Division of Pharmacy & Optometry, School of Health Sciences, Faculty of Biology, Medicine & Health, University of Manchester, Manchester, M13 9PT UK; 3 Faculté de Pharmacie, Université d’Auvergne, 63000 Clermont-Ferrand, France; 4 Wolfson Brain Imaging Centre, Department of Clinical Neurosciences, University of Cambridge, Box 65, Addenbrooke’s Hospital, Cambridge, CB2 0QQ UK

**Keywords:** ^11^C, Radiochemistry, PET, Methyl isocyanate, Carbamoylation

## Abstract

*N*-Methyl carbamoylimidazole is a safe and practical alternative to methyl isocyanate for carbamoylation reactions. We have developed a new chemical route for its synthesis from methyl iodide and applied this to the synthesis of *N*-[^11^C]methyl carbamoylimidazole as a new [^11^C]synthon to radiolabel biomolecules for PET imaging research. *N*-[^11^C]methyl carbamoylimidazole was prepared from [^11^C]methyl iodide in 70–74% radiochemical yield (decay corrected) and can be used in situ for further reaction without purification. The reactivity of *N*-[^11^C]methyl carbamoylimidazole was demonstrated in a series of [^11^C]carbamoylation reactions.

**Electronic supplementary material:**

The online version of this article (10.1007/s10967-018-5948-4) contains supplementary material, which is available to authorized users.

## Introduction

Methyl isocyanate [1] is a valuable chemical intermediate best known for its industrial synthesis applications [2]. Its chemistry is dominated by nucleophilic addition across the N=C bond to form *N*-methyl carbamoyl derivatives [3, 4]. Reactions with amines, alcohols and thiols to form the corresponding ureas, carbamates and thiocarbamates are the simplest examples of this type of chemistry which has been utilised to great effect to generate medicinally and biologically active products [5–8]. Within ^11^C radiochemistry for PET, [^11^C]methyl isocyanate is perhaps one of the lesser known radiolabelling synthons. However, with the option of being radiolabelled in the *N*-methyl or carbonyl position, its chemical versatility has allowed the creation of PET radiotracers across clinically important disease areas. It was first synthesised, albeit in low radiochemical yield, by a Curtis rearrangement reaction using [^11^C-*carbonyl*]acetyl chloride treated with tetrabutylammonium azide [9]. A subsequent improved radiochemical approach, the [^11^C]phosgenation of *N,N*-bis(trimethylsilyl)methylamine, gave [^11^C-*carbonyl*]methyl isocyanate in sufficient radiochemical yield to radiolabel physostigmine, a reversible acetylcholinesterase inhibitor [10]. In contrast, radiolabelling of methyl isocyanate in the *N*-methyl positon is achieved by the rearrangement reaction between [^11^C]methyl iodide and silver cyanate. [^11^C-*methyl*]Methyl isocyanate has been used to radiolabel the antitumour drug temozolomide in the *N*-methyl group of the tetrazinone ring [11].

*N*-Methyl carbamoylimidazole has now been developed as an alternative to methyl isocyanate for use in laboratory research [12]. It is water stable and has a similar reactivity profile to methyl isocyanate in converting primary and secondary amines to *N*-methylureas [5–8, 13]. The reactions are safe, rapid and in many cases give the resulting *N*-methylureas in quantitative yields. We present a new radiolabelling approach to *N*-[^11^C]methyl carbamoylimidazole (**1**) as a new [^11^C]synthon and demonstrate its reactivity towards a series of N, O and S nucleophiles.

### Materials and methods

#### Materials

*N*-Methyl carbamoylimidazole was purchased from Key Organics Ltd., Camelford, Cornwall, UK. All other chemicals were purchased from Sigma Aldrich Chemical Co., Gillingham, UK.

#### NMR spectra

NMR spectra were recorded using a Bruker Avance-400 spectrometer equipped with a 5 mm single-axis Z-gradient quattro nucleus probe, operating at 100 MHz for ^13^C NMR. The spectrometer was operated with TOPSPIN NMR software (Version 2.0). Chemical shifts (*δ*) are reported in parts per million (ppm), relative to (CH_3_)_4_Si (0.00 ppm) for ^13^C-NMR spectra.

#### *N*-Methyl carbamoylimidazole

^1^H NMR (400 MHz, DMSO-d_6_) *δ* 8.45 (br s, 1H), 8.21(br s, 1H), 7.64 (t, *J* = 1.3 Hz, 1H), 7.01 (br s, 1H), 2.81 (d, *J* = 4.5 Hz, 3H); ^13^C NMR (100 MHz, DMSO-d_6_) *δ* 149.6, 136.2, 130.0, 116.9, 27.2;

#### 1-Naphthyl-*N*-methylcarbamate

^1^H NMR (400 MHz, DMSO-d_6_) *δ* 8.09–8.04 (m, 1H), 8.03–7.91 (m, 1H), 7.88 (d, *J* = 7.8 Hz, 1H), 7.68–7.61 (m, 2H), 7.58 (t, *J* = 7.8 Hz, 1H), 7.35 (d, *J* = 7.8 Hz, 1H), 3.02 (d, *J* = 4.5 Hz, 0.3H), 2.80 (d, *J* = 4.5 Hz, 2.7H); ^13^C NMR (100 MHz, DMSO-d_6_) *δ* 155.5, 147.8, 134.6, 127.9, 127.4, 126.3, 126.2, 125.5, 125.4, 121.3, 119.1, 27.7.

#### *S*-(4-Chlorophenyl) *N*-methylthiocarbamate

^1^H NMR (400 MHz, DMSO-d_6_) *δ* 7.47 (br s, 4H), 5.70 (br s, 1H), 2.67 (d, *J* = 4.5 Hz, 3H); ^13^C NMR (100 MHz, DMSO-d_6_) *δ* 165.8, 136.5, 134.5, 129.5, 126.8, 28.1.

#### 1-(Benzo[d]thiazol-2-yl)-3-methylurea

^1^H NMR (400 MHz, DMSO-d_6_) *δ* 10.87 (br s, 1H), 7.84 (dd, *J* = 8.0, 1.0 Hz, 1H), 7.60 (br d, *J* = 8.0 Hz, 1H), 7.35 (td, *J* = 7.5, 1.0 Hz, 1H), 7.20 (td, *J* = 7.5, 1.0 Hz, 1H), 6.77 (br s, 1H), 2.73 (d, *J* = 4.5 Hz, 3H).

#### Radio-HPLC chromatography

Radio-HPLC analysis was carried out on a Shimadzu prominence system consisting of a CBM-20A controller, a LC-20AB solvent delivery system, SPD-20A absorbance detector and a radio-HPLC Bioscan Flowcount B-FC 3100 detector. The system was operated using LabLogic software (Laura 3.0).

#### Automated radiochemistry system

The automated radiochemistry system was a Tracerlab FX-FE module manufactured by GE Healthcare. The system was modified by the insertion of a Carbolite MTF 9/15 tube furnace, operating at 180 °C, in between Valve 1 and Valve 2 (Fig. 3). Prior to the start of each synthesis, reagents were either added directly to Reaction Vessel A or loaded into Reagent Vials A and B then transferred to Reaction Vessel A under helium gas pressure during the synthesis.

### Synthesis of [^11^C]methyl iodide

[^11^C]Methyl iodide was prepared from cyclotron produced [^11^C]carbon dioxide using a GE PETtrace MeI Microlab radiochemistry system. The synthesis time from the end of cyclotron target bombardment (EOB) was 12 min. A full account of the method has been previously reported [14, 15]. For the work described here, [^11^C]methyl iodide is delivered under a flow of helium to an automated radiochemistry system (Fig. 3).

### Synthesis of [^11^C-*methyl*]methyl isocyanate from [^11^C]methyl iodide

[^11^C]Methyl isocyanate was prepared from [^11^C]methyl iodide using an automated radiochemistry system (Fig. 3) fitted with a Carbolite MTF 9/15 tube furnace (18 × 1.5 cm) having adjustable temperature control. A full account of the method has been previously reported [11]. To summarise, [^11^C]methyl iodide was distilled under nitrogen (10 mL min^−1^) across silver cyanate (250 mg, 1.67 mmol) at 180 °C for 1 min and the products were trapped in Reaction Vessel A (Fig. 3) containing anhydrous acetonitrile (250 μL). Typically, the conversion of [^11^C]methyl iodide to [^11^C]methyl isocyanate was 70–75% (decay corrected). The total radiosynthesis time from EOB was 13 min (this included 12 min for [^11^C]methyl iodide production).

### Synthesis of *N*-[^11^C]methyl carbamoylimidazole (**1**) from [^11^C-*methyl*]methyl isocyanate

Vaporised [^11^C]methyl isocyanate was passed into a solution of imidazole (2 mg) in acetonitrile (250 μL) which was preloaded into Reaction Vessel A of the automated radiochemistry system (Fig. 3). The reaction mixture was sealed and stirred at 80 °C for 5 min then cooled to 25 °C. The total radiosynthesis time from EOB to obtain the cooled solution was 19 min (this included 13 min for [^11^C]methyl isocyanate production). For analysis, a 1.0 mL mixture of water:acetonitrile (65:35, v/v) was added to Reaction Vessel A from Reagent Vial A (Fig. 3) and the resultant mixture was loaded onto a HPLC column [Prodigy C18 ODS(3), 10 µm particle size 250 × 10 mm i.d]. The column was eluted at a flowrate of 3 mL min^−1^ using a mixture of water:acetonitrile (65:35, v/v). The mobile phase was monitored for both radioactivity and UV absorbance at 254 nm. The fraction eluting between 7 and 8 min, having the same retention time as reference *N*-methyl carbamoylimidazole (7.33 min) was collected.

### Synthesis of 1-naphthyl-*N*-[^11^C]methylcarbamate (**2**) from *N*-[^11^C]methyl carbamoylimidazole (**1**)

A 250 μL solution of *N*-[^11^C]methyl carbamoylimidazole (**1**) in acetonitrile was first prepared in Reaction Vessel A of the automated radiochemistry system (Fig. 3) as described earlier. Once prepared, it was immediately used in situ by the following procedure. A solution of 1-naphthol (2 mg) in acetonitrile (300 μL) containing triethylamine (10 µL) was added to Reaction Vessel A from Reagent Vial A and the reaction mixture stirred at 65 °C for 3 min. After cooling to 25 °C, a 1.0 mL mixture of water:ethanol (55:45, v/v) was added to Reaction Vessel A from Reagent Vial B and the resultant mixture was loaded onto a HPLC column [Prodigy C18 ODS(3), 10 µm particle size, 250 × 10 mm i.d]. The column was eluted at a flowrate of 3 mL min^−1^ using a mixture of water:ethanol (55:45, v/v). The mobile phase was monitored for both radioactivity and UV absorbance at 254 nm. The fraction eluting between 12 and 13 min, having the same retention time (12.33 min) as reference 1-naphthyl-*N*-methylcarbamate was collected. The total radiosynthesis time from EOB was 37 min (this included 19 min for production of *N*-[^11^C]methyl carbamoylimidazole).

### Synthesis of *S*-(4-chlorophenyl) *N*-[^11^C]methylthiocarbamate (**3**) from *N*-[^11^C]methyl carbamoylimidazole (**1**)

A 250 μL solution of *N*-[^11^C]methyl carbamoylimidazole (**1**) in acetonitrile was first prepared in Reaction Vessel A of the automated radiochemistry system (Fig. 3) as described earlier. Once prepared, it was immediately used in situ by the following procedure. A solution of 4-chlorophenylthiophenol (2 mg) in acetonitrile (300 μL) containing triethylamine (10 µL) was added to Reaction Vessel A from Reagent Vial A and the reaction mixture stirred at 65 °C for 3 min. After cooling to 25 °C, a 1.0 mL mixture of water:acetonitrile (60:40, v/v) containing 0.1% trifluoroacetic acid was added to Reaction Vessel A from Reagent Vial B and the resultant mixture was loaded onto a HPLC column (ACE 3 Phenyl, 10 µm particle size, 150 × 4.6 mm i.d). The column was eluted at a flowrate of 1 mL min^−1^ using a mixture of water:acetonitrile (60:40, v/v) containing 0.1% trifluoroacetic acid. The mobile phase was monitored for both radioactivity and UV absorbance at 254 nm. The fraction eluting between 7 and 8 min, having the same retention time (7.23 min) as reference *S*-(4-chlorophenyl) *N*-methylthiocarbamate was collected. The measured molar activity at the end of radiosynthesis was 59–69 GBq μmol^−1^ and the total radiosynthesis time from EOB was 32 min (this included 19 min for production of *N*-[^11^C]methyl carbamoylimidazole).

### Synthesis of 1-(benzo[d]thiazol-2-yl)-3-[^11^C]methylurea (**4**) from *N*-[^11^C]methyl carbamoylimidazole (**1**)

A 250 μL solution of *N*-[^11^C]methyl carbamoylimidazole (**1**) in acetonitrile was first prepared in Reaction Vessel A of the automated radiochemistry system (Fig. 3) as described earlier. Once prepared, it was immediately used in situ by the following procedure. A solution of 2-aminobenzothiazole (2.0 mg) in acetonitrile (300 μL) containing sodium hydride (1.0 mg) was added to Reaction Vessel A from Reagent Vial A and the reaction mixture stirred at 65 °C for 3 min. After cooling to 25 °C, a 1.0 mL mixture of water:ethanol (65:35, v/v) was added to Reaction Vessel A from Reagent Vial B and the resultant mixture was loaded onto a HPLC column [Prodigy C18 ODS(3), 10 µm particle size, 250 × 10 mm i.d.]. The column was eluted at a flowrate of 3 mL min^−1^ using a mixture of water:ethanol (65:35, v/v). The mobile phase was monitored for both radioactivity and UV absorbance at 254 nm. The fraction eluting between 12 and 14 min, having the same retention time (12.90 min) as reference 1-(benzo[d]thiazol-2-yl)-3-methylurea was collected. The total radiosynthesis time from EOB was 38 min (this included 19 min for production of *N*-[^11^C]methyl carbamoylimidazole).

## Results and discussion

### Radiosynthesis of *N*-[^11^C]methyl carbamoylimidazole (**1**)

**Fig. 1:**
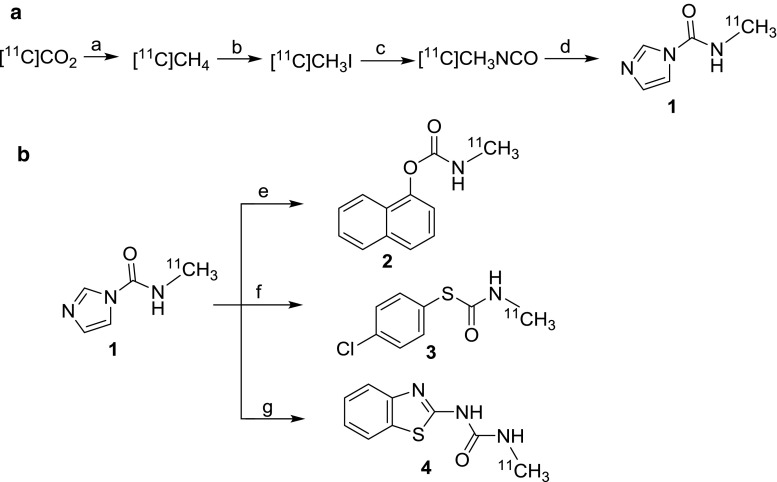
Radiosynthesis and reactivity of *N*-[^11^C]methyl carbamoylimidazole: (a) Ni, hydrogen, 380 °C, 4 min; (b) iodine, 720 °C, 8 min; (c) silver cyanate, 180 °C, 1 min; (d) imidazole, dry ACN, 80 °C, 5 min; (e) 1-napthol, triethylamine, dry ACN, 65 °C, 3 min; (f) 4-chlorothiophenol, triethylamine, dry ACN, 65 °C, 3 min; (g) 2-aminobenzothiazole, sodium hydride, dry ACN, 65 °C, 3 min

**Fig. 2:**
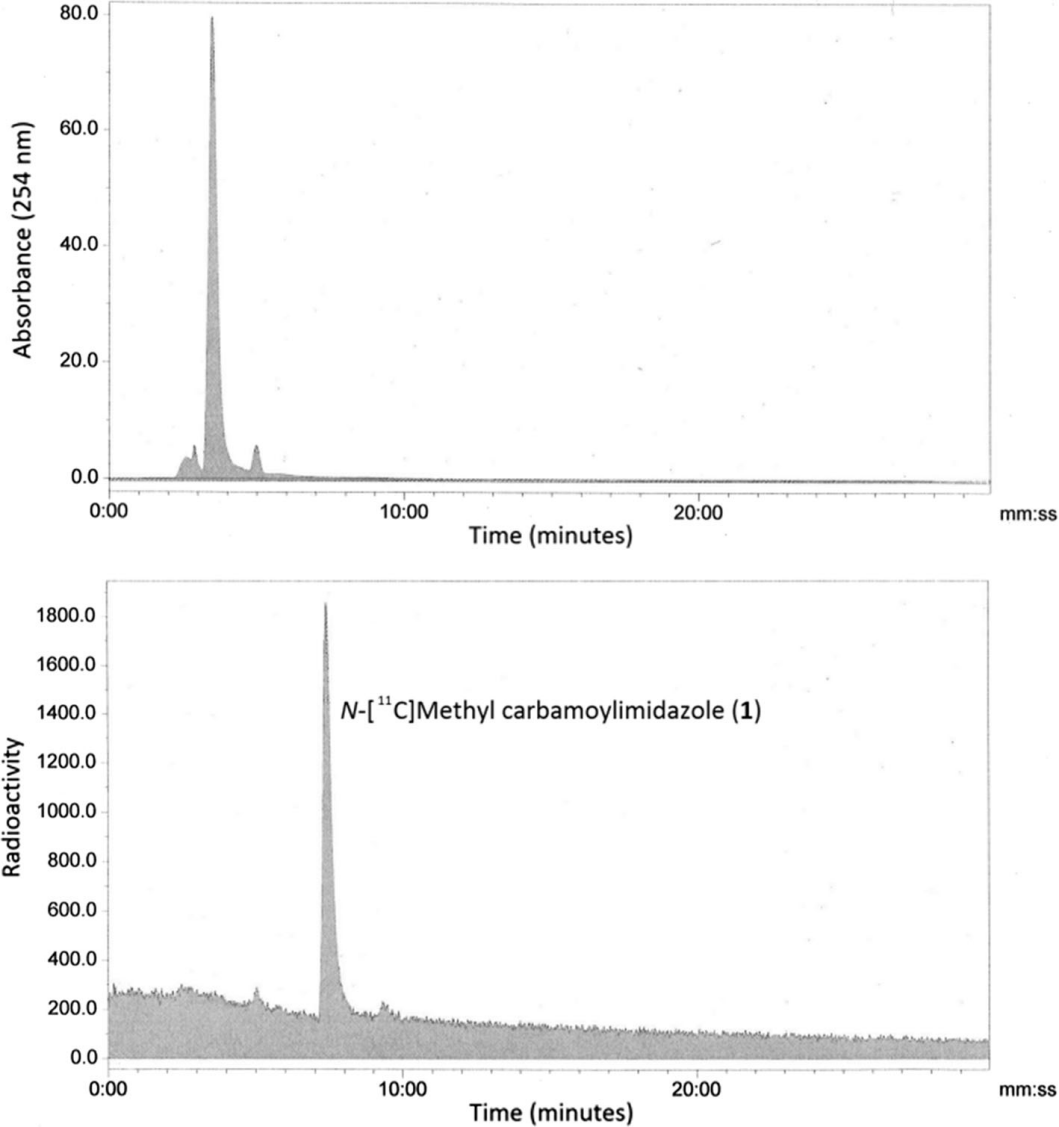
A typical HPLC chromatogram from the synthesis of *N*-[^11^C]methyl carbamoylimidazole (**1**)

In our previous work we described the radiosynthesis of a range of [^11^C]organo isocyanates and their applications in addition or cycloaddition reactions across the isocyanate C=N bond [11, 16]. Herein we describe for the first time the radiosynthesis of *N*-[^11^C]methyl carbamoylimidazole (**1**) as a water stable alternative to [^11^C]methyl isocyanate. The radiosynthesis was carried out by first preparing [^11^C]methyl isocyanate from [^11^C]methyl iodide (Fig. 1A). This involved the production and flow of vaporised [^11^C]methyl iodide across silver cyanate contained in a tube furnace at 180 °C. The [^11^C]methyl isocyanate outflow was trapped in a solution containing imidazole in acetonitrile and the resulting mixture heated under stirred conditions to form *N*-[^11^C]methyl carbamoylimidazole. For analysis, the reaction mixture was diluted with HPLC eluent and loaded onto a semi-preparative HPLC column from which the eluent was monitored for radioactivity and absorbance. A single radioactive peak with the same retention time (7.33 min) as authentic *N*-methyl carbamoylimidazole was observed and accounted for > 95% of the injected radioactivity (Fig. 2). No degradation in the radioactivity profile was observed when the aqueous reaction mixture was analysed under room temperature conditions at 20, 40 and 80 min timepoints after completion of the radiosynthesis. Conversion to *N*-[^11^C]methyl carbamoylimidazole based on the starting amount of [^11^C]methyl iodide was 70–74% (decay corrected, *n* = 6) with a total radiosynthesis time of 19 min from the end of cyclotron target bombardment (EOB). The main loss in radiochemical yield (ca. 25%) was incurred during [^11^C]methyl isocyanate production due to radioactivity retained on the silver cyanate column that could not be recovered.

**Fig. 3:**
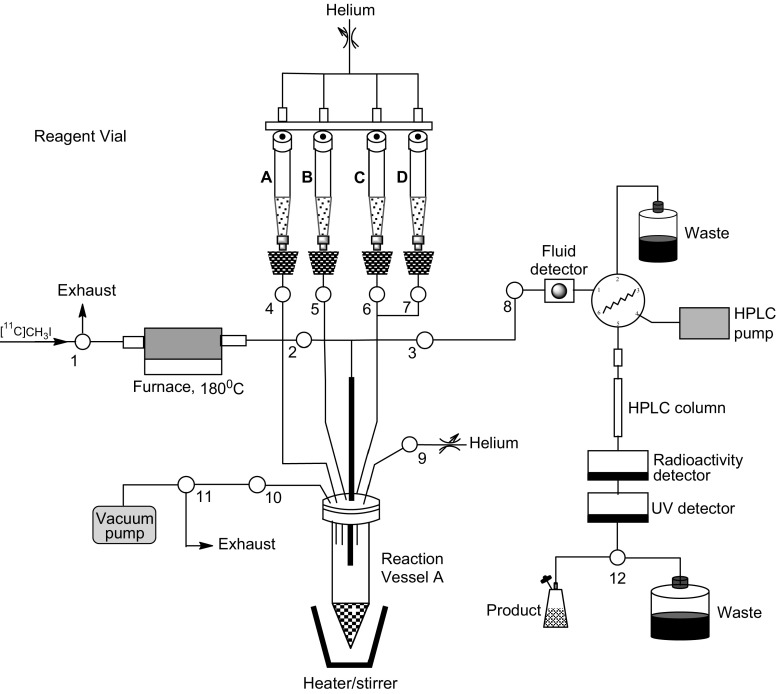
Automated radiochemistry system

**Fig. 4:**
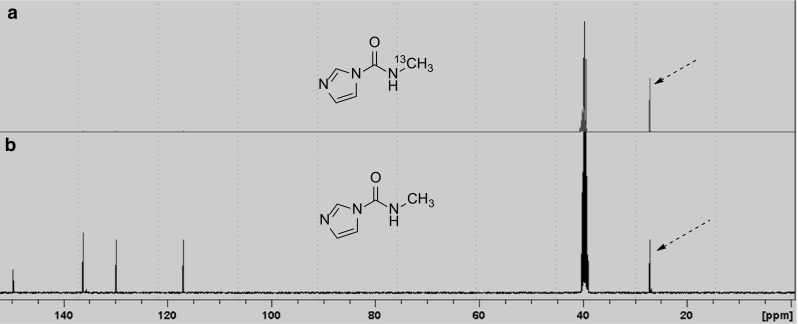
^13^C NMR spectra (d_6_-DMSO) of *N*-[^13^C]methyl carbamoylimidazole; **a** spectrum recorded after radioactive decay of *N*-[^11/13^C]methyl carbamoylimidazole; **b** spectrum of authentic *N*-methyl carbamoylimidazole (**1**). The dotted arrows denote the ^13^C-signal for the *N*-methyl group

A [^11^/^13^C]co-labelling experiment was performed to verify the radiosynthesis of *N*-[^11^C]methyl carbamoylimidazole (**1**) and confirm the position of labelling in the *N*-methyl position. This involved modification to the automated synthesis system (Fig. 3) by the insertion of a vial containing a small aliquot [^13^C]methyl iodide between Valve 1 and the heated silver cyanate salt. Initially, [^11^C]methyl iodide was prepared and then transferred under a flow of nitrogen into the small vial containing [^13^C]methyl iodide. Under the same nitrogen flow, the resultant [^11/13^C]methyl iodide mixture was transferred out of the vial and across silver cyanate at 180 °C to form [^11/13^C]methyl isocyanate. Reaction of [^11/13^C]methyl isocyanate with imidazole gave *N*-[^11/13^C]methyl carbamoylimidazole which was then isolated by semi-preparative radio-HPLC. After radioactive decay, the isolated product was examined by proton-decoupled ^13^C NMR spectroscopy (100 MHz). A single peak at 27.2 ppm was observed, having the same chemical shift as the *N*-methyl group of authentic *N*-methyl carbamoylimidazole (Fig. 4).

### Reactivity of *N*-[^11^C]methyl carbamoylimidazole (**1**)

**Fig. 5:**
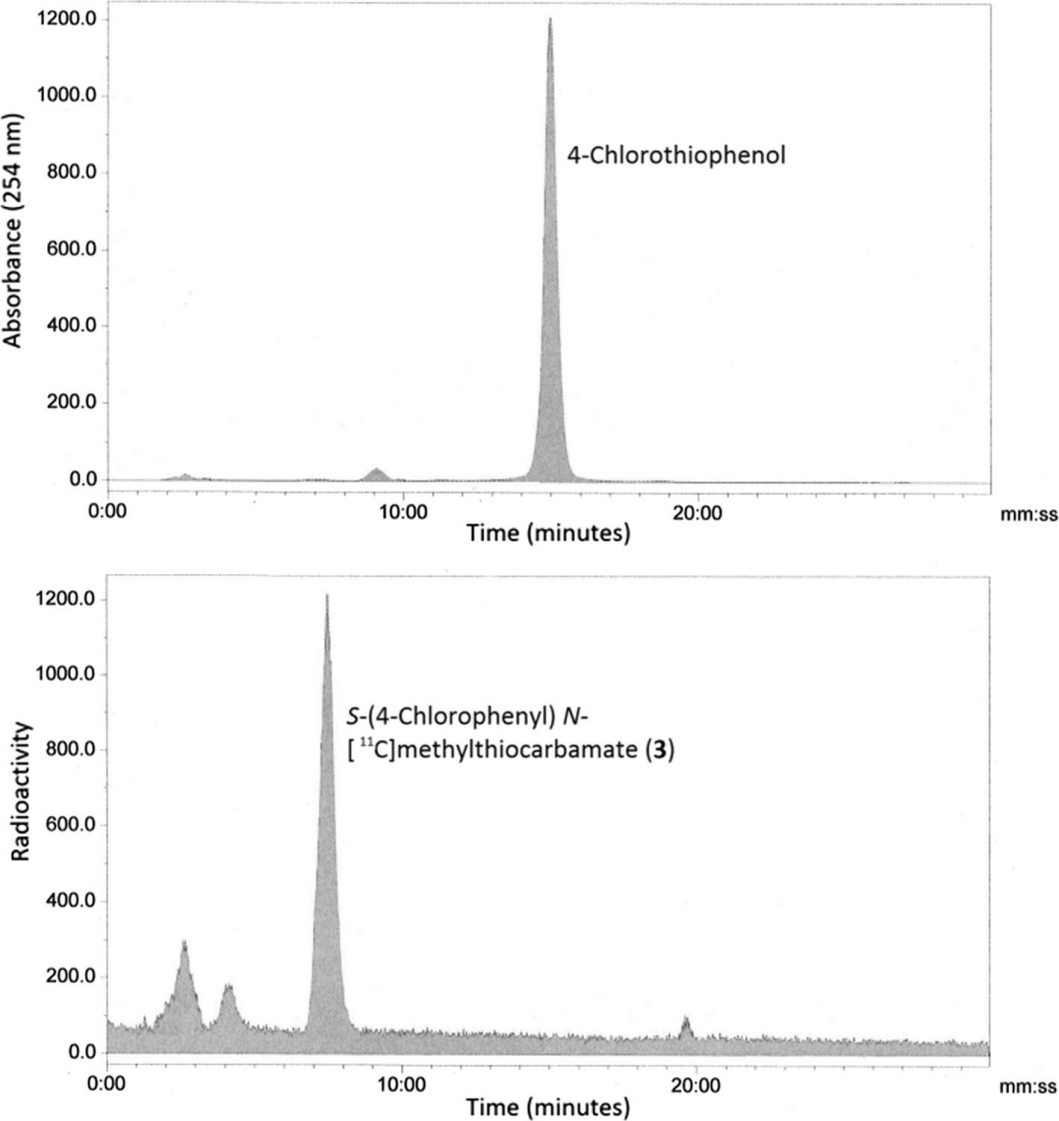
A typical HPLC chromatogram from the synthesis of *S*-(4-chlorophenyl) *N*-[^11^C]methylthiocarbamate (**3**)

**Fig. 6:**
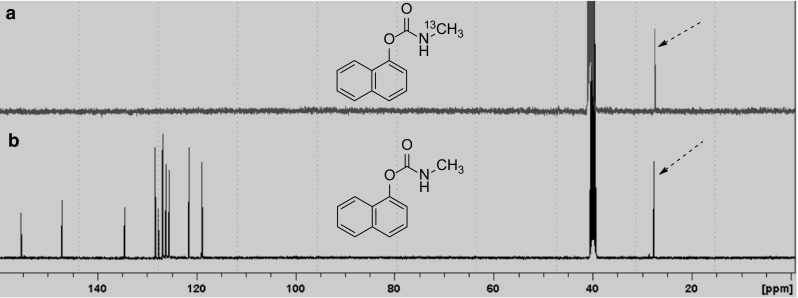
^13^C NMR (d_6_-DMSO) spectra of 1-naphthyl *N*-[^13^C]methylcarbamate: **a** spectrum recorded after radioactive decay of 1-naphthyl *N*-[^11/13^C]methylcarbamate; **b** spectrum of authentic 1-naphthyl *N*-[^13^C]methylcarbamate. The dotted arrows denote the ^13^C-signal for the *N*-methyl group

To demonstrate the reactivity of *N*-[^11^C]methyl carbamoylimidazole it was treated with N, O and S nucleophiles in a series of base-promoted [^11^C]carbamoylation reactions (Fig. 1B). For each reaction, *N*-[^11^C]methyl carbamoylimidazole was produced and used in situ with no further purification. 2-Aminobenzothiazole, 1-naphthol and 4-chlorothiophenol were chosen as the N, O and S nucleophiles respectively. They typically react rapidly at room temperature under specific solvent conditions [12]. However, for translation to an automated [^11^C]radiochemical synthesis the conditions had to be modified to achieve compatibility with the *N*-[^11^C]methyl carbamoylimidazole synthesis method. All reactions were therefore performed in acetonitrile and under heated conditions. The reactions were analysed by radio-HPLC and in each case the retention time of the main radioactive product was the same as that of an authentic reference of the corresponding *N*-[^11^C]methyl carbamoylated adduct (Fig. 5). Thus 1-naphthyl-*N*-[^11^C]methylcarbamate (**2**), *S*-(4-chlorophenyl) *N*-[^11^C]methylthiocarbamate (**3**) and 1-(benzothiazol-2-yl)-3-[^11^C]methylurea (**4**) were all synthesised from [^11^C]methyl iodide via *N*-[^11^C]methyl carbamoylimidazole (**1**) as an intermediate synthon (Fig. 1b). Their radiosyntheses were complete in 37, 32 and 38 min from EOB respectively. Based on the starting amount of [^11^C]methyl iodide, 1-(benzothiazol-2-yl)-3-[^11^C]methylurea (**4**) and *S*-(4-chlorophenyl) *N*-[^11^C]methylthiocarbamate (**3**) were produced in moderate radiochemical yields (decay corrected) of 71 and 64% respectively (*n* = 3 for both), whereas for 1-naphthyl-*N*-[^11^C]methylcarbamate (**2**) the radiochemical yield was 18% (*n* = 3). It was noted that the radiochemical yields are lower than the reported corresponding chemical yields of 1-(benzothiazol-2-yl)-3-methylurea (94%), *S*-(4-chlorophenyl) *N*-methylthiocarbamate (76%) and 1-naphthyl-*N*-methylcarbamate (65%) from *N*-methyl carbamoylimidazole [12]. However, the radiochemical yields do follow a similar pattern which perhaps reflects the relative nucleophilicity of the respective precursor compounds. Finally, to demonstrate the method of radiolabelling by [^11^C]carbamoylation, 1-napthol was used in a [^11/13^C]carbamoylation co-labelling reaction in the same manner as was used to verify the radiosynthesis of *N*-[^11^C]methyl carbamoylimidazole (**1**). The isolated reaction product was examined by proton-decoupled ^13^C NMR (100 MHz) and a single peak at 27.7 ppm was observed having the same chemical shift as the *N*-methyl group of authentic 1-naphthyl-*N*-methylcarbamate (Fig. 6).

## Conclusions

*N*-Methyl carbamoylimidazole is a useful alternative to methyl isocyanate for carbamoylation reactions. This is particularly true under aqueous conditions where methyl isocyanate is readily hydrolysed to 1,3-dimethylurea [17] whereas *N*-methyl carbamoylimidazole is highly stable and retains its reactivity towards N, O and S nucleophiles. A fast and reproducible method has been developed for the radiosynthesis of *N*-[^11^C]methyl carbamoylimidazole (**1**) from [^11^C]methyl iodide in good radiochemical yield. We have shown that it is stable in aqueous-based media and demonstrated its reactivity in a series of [^11^C]carbamoylation reactions with N, O and S nucleophiles. Both the synthesis of *N*-[^11^C]methyl carbamoylimidazole (**1**) and its reactivity have been confirmed in [^11/13^C]co-labelling experiments using [^13^C]methyl iodide. Under the method conditions developed, *N*-[^11^C]methyl carbamoylimidazole (**1**) reacted moderately well with N and S nucleophiles in comparison to an O nucleophile.

## Electronic supplementary material

Below is the link to the electronic supplementary material.

Supplementary material 1 (DOCX 2285 kb)
