# Supplementary material for: Radiosynthesis and reactivity of N-[11C]methyl carbamoylimidazole
Source: J Radioanal Nucl Chem. 2018 Jun 19;317(2):977–84. doi: 10.1007/s10967-018-5948-4 (PMC6061098; doi:10.1007/s10967-018-5948-4)
Supplement: Supplementary file 1 — Supplementary material 1 (DOCX 2285 kb) [file 10967_2018_5948_MOESM1_ESM.docx]

1 Radio-HPLC analysis of 1-naphthyl-*N*-[^11^C]methylcarbamate (2)


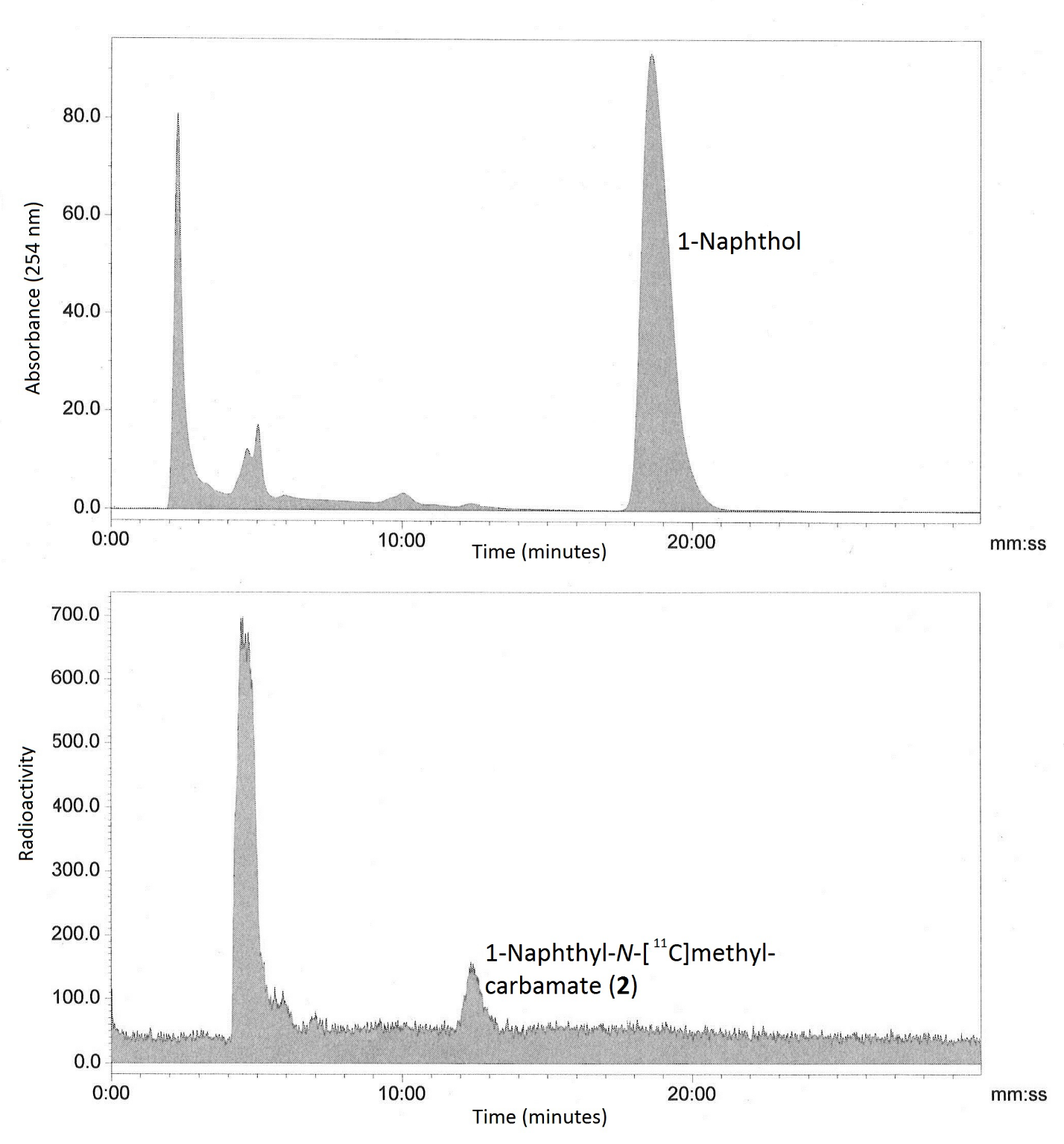


HPLC column: Phenomenex Prodigy C18 ODS(3), 10 µm particle size, 250 x 10 mm i.d.

Eluent: water: ethanol (55:45, v/v)

Flow rate: 3 mL min^-1^

UV wavelength: 254 nm

2 Radio-HPLC analysis of 1-(benzo[d]thiazol-2-yl)-3-[^11^C]methylurea (4)


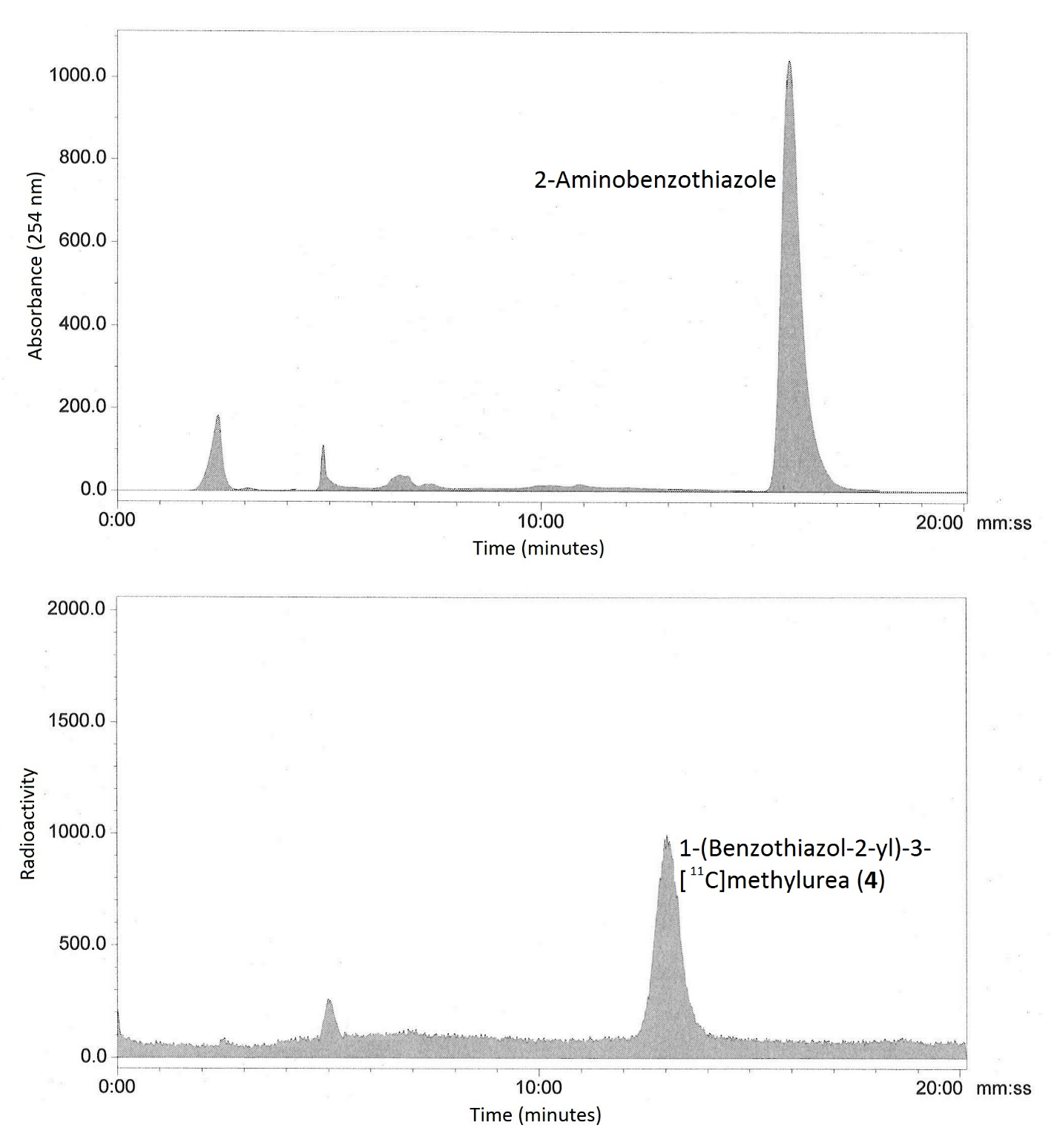


HPLC column: Phenomenex Prodigy C18 ODS(3), 10 µm particle size, 250 x 10 mm i.d.

Eluent: water: ethanol (65:35, v/v)

Flow rate: 3 mL min^-1^

UV wavelength: 254 nm

3 HPLC analysis of 1-naphthyl-*N*-methylcarbamate


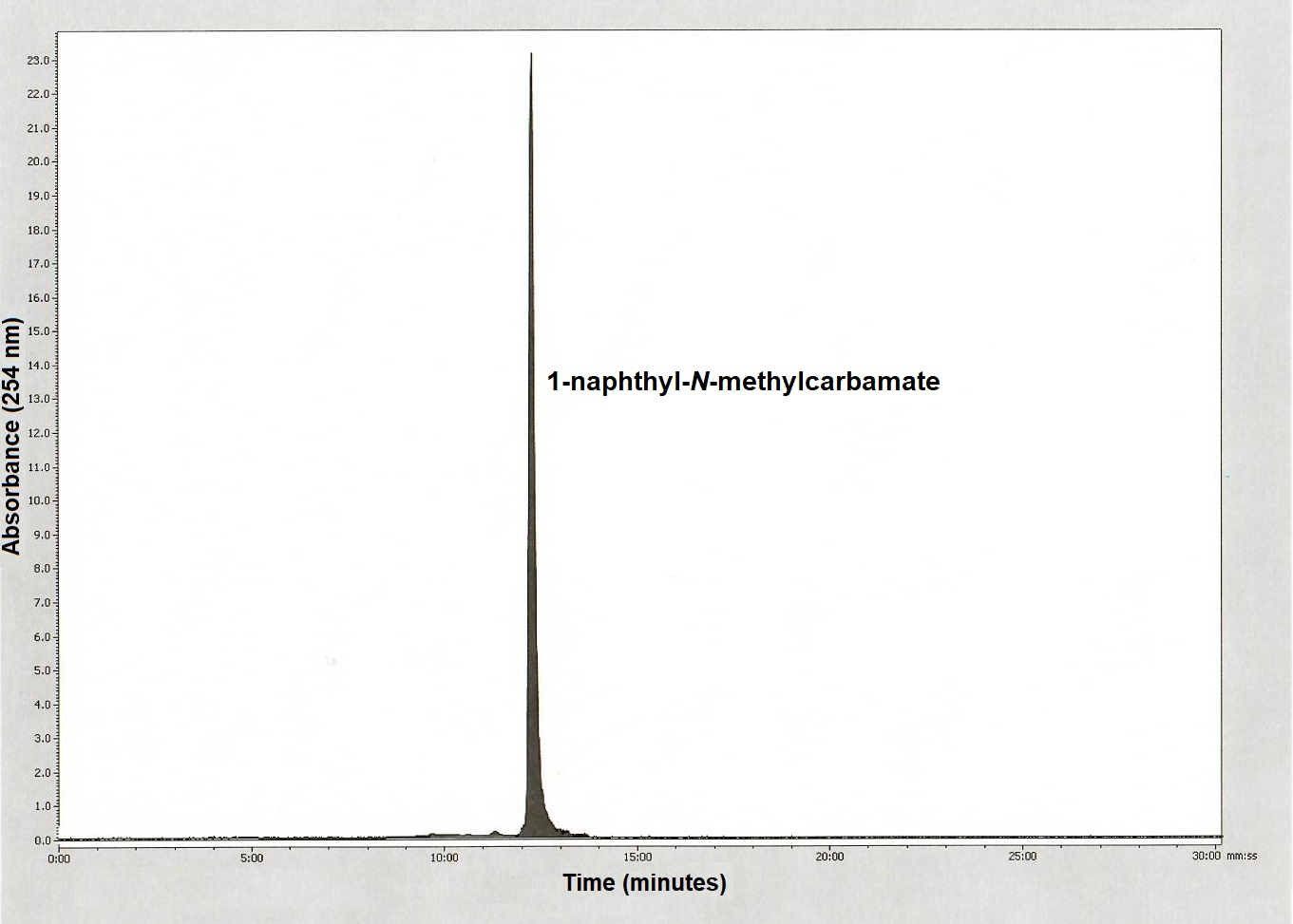


HPLC column: Phenomenex Prodigy C18 ODS(3), 10 µm particle size, 250 x 10 mm i.d.

Eluent: water: ethanol (55:45, v/v)

Flow rate: 3 mL min^-1^

UV wavelength: 254 nm

Sample concentration: 100 μg mL^-1^

4 HPLC analysis of *S*-(4-chlorophenyl) *N*-methylthiocarbamate


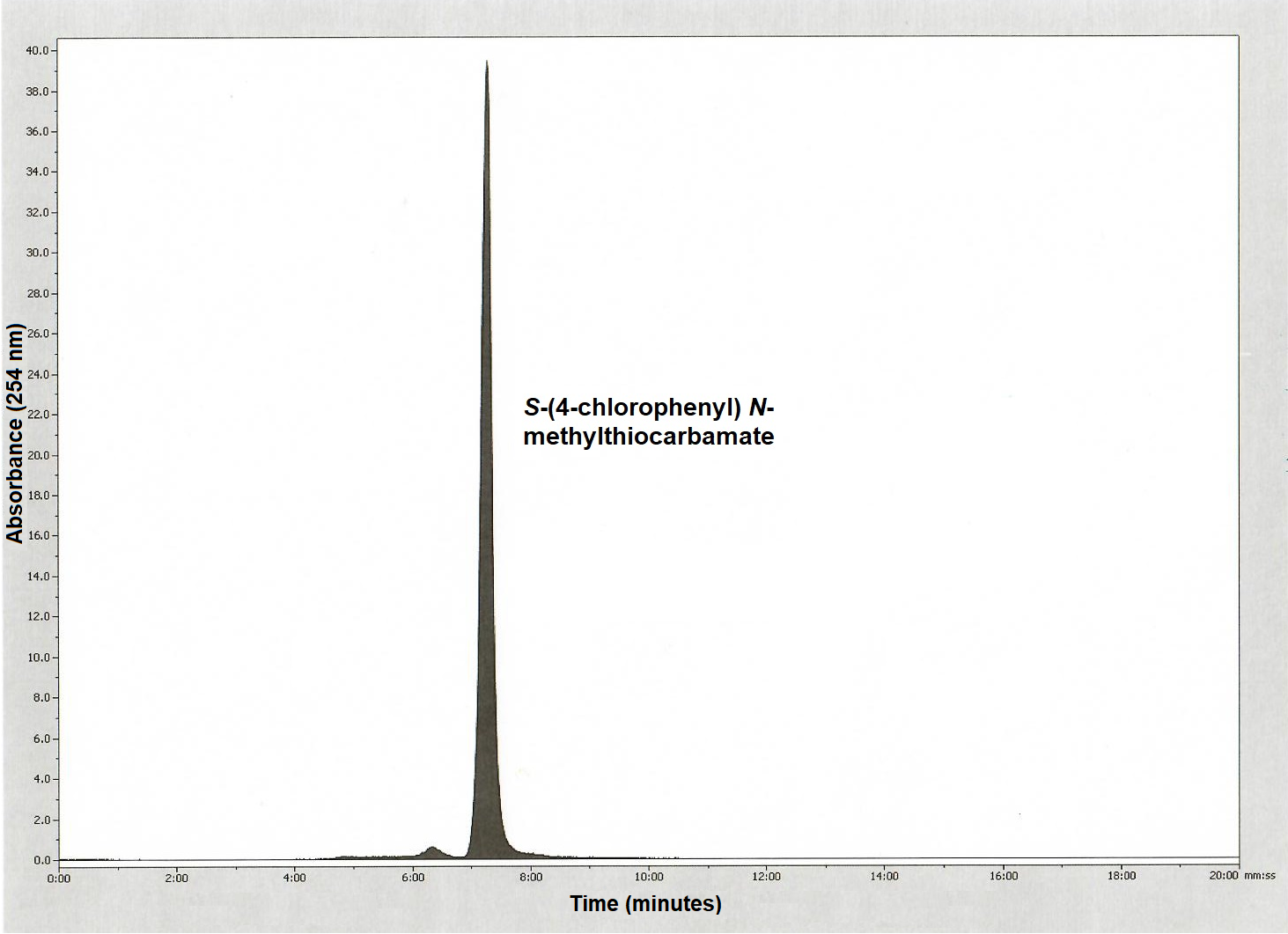


HPLC column: ACE 3 Phenyl, 10 µm particle size, 150 x 4.6 mm i.d

Eluent: water: acetonitrile (60:40, v/v) containing 0.1% trifluoroacetic acid

Flow rate: 1 mL min^-1^

UV wavelength: 254 nm

Sample concentration: 100 μg mL^-1^

5 HPLC analysis of 1-(benzo[d]thiazol-2-yl)-3-methylurea


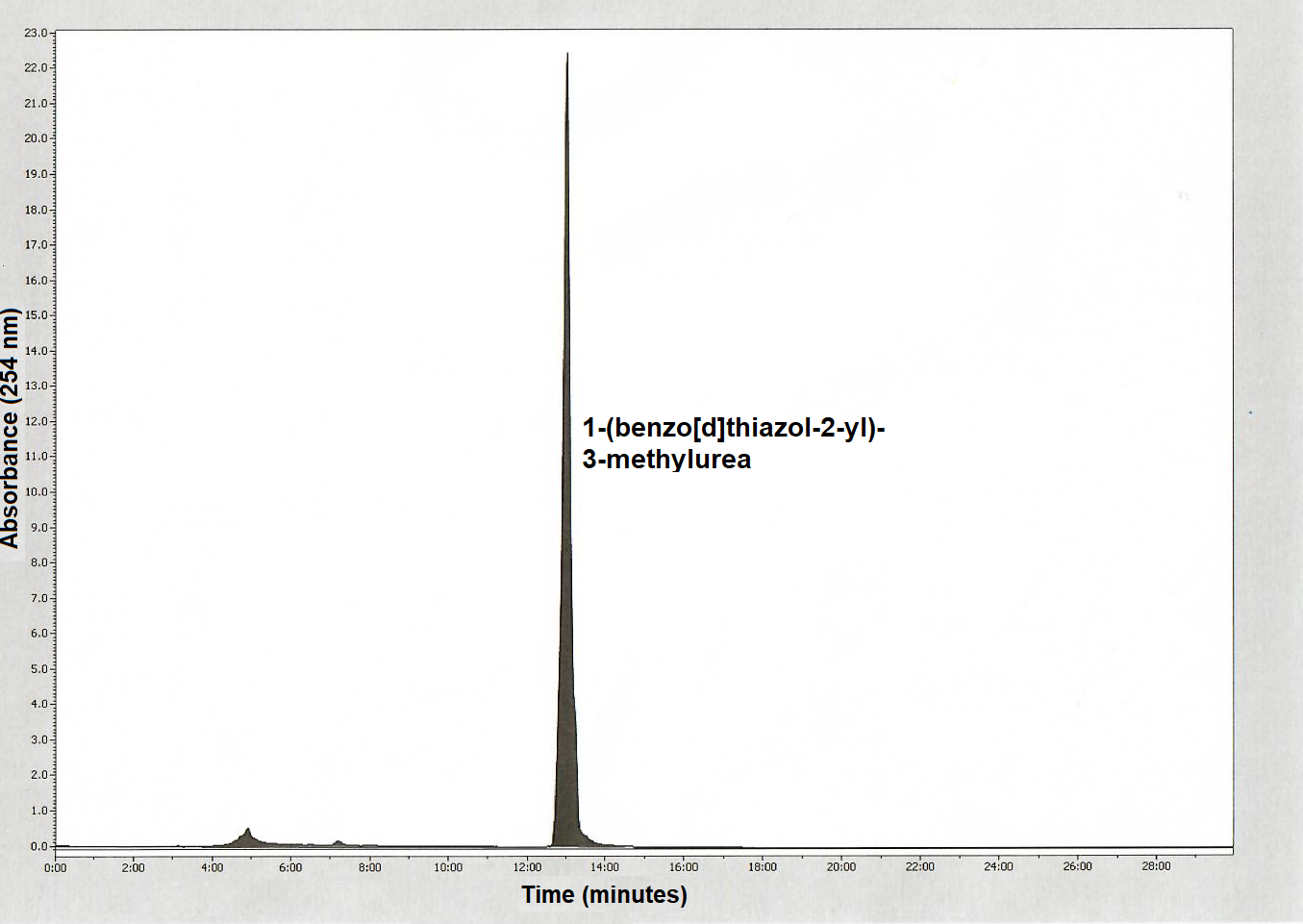


HPLC column: Phenomenex Prodigy C18 ODS(3), 10 µm particle size, 250 x 10 mm i.d.

Eluent: water: ethanol (65:35, v/v)

Flow rate: 3 mL min^-1^

UV wavelength: 254 nm

Sample concentration: 100 μg mL^-1^
